# Assessment role of total phenols and flavonoids extracted from *Pleurotus columbinus* mushroom on the premature ovarian failure induced by chemotherapy in rats

**DOI:** 10.1186/s43141-021-00278-0

**Published:** 2021-12-10

**Authors:** Amal I. Hassan, Mona A. M. Ghoneim, Manal G. Mahmoud, Mohsen S. Asker

**Affiliations:** 1 Department of Radioisotopes, Nuclear Research Centre, Egyptian Atomic Energy Authority, Cairo, Egypt; 2 Microbial Biotechnology Department, National Research Centre, Dokki, Giza, Egypt

**Keywords:** Cyclophosphamide, Mushroom, Flavonoids, Total phenols, Natural antioxidant

## Abstract

**Background:**

Many species of mushroom contain an assortment of free radical scavengers (Phenolics and Flavonoids compounds) that have made them nutritionally beneficial and a source of expansion of drug production. In this study, we examined the preventive and remedial role of total phenol extract from *Pleurotus columbines* (TP) in alleviating the consequences of cyclophosphamide (CTX) on the ovaries of female rats. Rats were randomly assigned to four groups: healthy controls, cyclophosphamide (CTX), received a TP (100 mg/kg) orally daily for 14 days and curative group: CTX-TP, we determined and identified a total phenol from a mushroom extract and examined it as an antioxidant agent. To investigate the therapeutic influence, it was administrated 2 weeks after CTX. To assess the impact of TP on ovarian damage caused by CTX, ovarian hormone tests were performed such as luteinizing hormone (LH), 17-β-estradiol (E2), and anti-mullerian hormone (AMH). Besides, follicle-stimulating hormone (FSH) in serum was evaluated, and histopathological analysis of the ovary was examined.

**Results:**

This study indicates that treatment with TP decreased the severity of cyclophosphamide-induced ovary injury by reducing inflammation and apoptotic effects and increasing the activity of antioxidants.

**Conclusions:**

TP could be used to alleviate cyclophosphamide-induced ovary injury.

## Background

Mushrooms such as *Pleurotus* sp. are considered therapeutic agents for over 100 years. Based on the presence of proteins, carbohydrates, vitamins, minerals, essential unsaturated fatty acids, aromatic phenols, terpenoids, steroids, and water content, one of these mushrooms has the highest biological nutritional value [1]. The *Pleurotus* has many active metabolites, which are sources of pharmaceuticals for several diseases, including hypertension, anemia, diabetes, obesity, inflammatory, viral, mutagenic, and oxidant diseases [2]. The mushroom phenolics include benzoicacid, trans-3, 4-dihydride-3, 4, 8-trihydroxide, 1(2H)-one, 4-hydroxy benzaldehyde, and the flavonoids include quercetin, catechin, and chrysin (Papaspyrid). Besides dietary fibers, chitin, and glucans, mushrooms contain intrinsic cell-walls constituents such as riboflavin and other B-group vitamins, selenium, copper, and potassium, also dietary fibers, chitin, and potassium [3]. While mushrooms are the best dietary source of the extraordinary sulfur-containing antioxidants ergothioneine, the active antioxidant “glutathione” is evaluated [4]. Recently, acute dosing with CTX was shown to reduce ovarian follicles over 3 days in Sprague-Dawley rats [5]. Besides, an inhibitory effect of cyclophosphamide has been detected on the hypothalamic-pituitary axis in rhesus monkeys [6]. This finding shows ovarian failure itself as excessive gonadal insufficiency, leading to menopause and usually irreversible sterility. When all follicles containing the eggs are damaged, the chance of pregnancy becomes impossible. Various laboratory investigations have isolated compounds with benefits for humans from several mushrooms. Lee et al. [7] stated that eating mushrooms eliminates epithelial ovarian cancer in women in southern China, as this fungus protects against this type of cancer. Therefore, the present study was intended to appraise the antioxidant activity of an aqueous extract of *P. columbinus* and its impact on premature ovarian failure (POF) triggered by chemotherapy in rats.

## Methods

### Mushroom sample collection

A sample of dry *Pleurotus columbinus* was purchased from the Microbiological Culture Center (Cairo, Egypt) and used throughout the study period.

### Extraction of mushroom

Dried powdered fruiting bodies (20 g) were extracted by stirring with 200 mL distilled water for 24 h and then centrifuged at 5000×*g* for 30 min using 2K15 Sigma-Laborzentrifugen (Germany), and the supernatant was stored at −20 °C. The residue was re-extracted with 30 ml distilled water; the combined aqueous extracts were lyophilized.

### Mushroom composition

The contents of crude protein, raw fiber, crude fat, and ash of the mushroom were assayed according to Association of Official Analytical Chemists (AOAC) methods. In addition, mushroom polysaccharide content was calculated using the phenol-sulfuric acid method of Lu et al. [8].

### Determination of total phenolic content

The total phenolic content was determined using the spectrophotometer technique according to Li et al. [9]. First, the dried extract was dissolved in distilled water (1 mg/mL) and then 0.5 mL was added to 2.5 mL of 10% Folin-Ciocalteu reagent and 2 mL of NaCO_3_ (2% w/v). The mixture was incubated at 45 °C with shaking for 15 min. Absorbance was measured at 765 nm using a UV/VIS spectrophotometer (UV-1700 pharam sec, Shimadzu, Japan), and the phenolic content was quantified regarding a standard curve of gallic acid.

### Estimation of total flavonoids

Flavonoids were assayed according to the colorimetric method of Li et al. [9]. To 1 mL of the mushroom extract, 3 mL methanol, 0.2 mL of 10% AlCl_3_, 0.2 mL of 1 M potassium acetate, and 5.6 mL distilled water were added. The mixture was incubated at room temperature for 30 min. The absorbance of the reaction mixture was measured at 415 nm using a UV/VIS spectrophotometer (UV-1700 pharma sec, Shimadzu, Japan). Quercetin was used to calculate the standard curve.

### Designating phenolics by high performance liquid chromatographic (HPLC)

The lyophilized mushroom extract was fractionated using liquid chromatography (Shimadzu corporation-Kyoto, Japan). This parathion was achieved at a reversed-phase C18 column (250 × 4.6 mm, 5 microns) temperature at 24 °C. The mobile phase of the optimized chromatographic method comprised solvent A (methanol) and solvent B 0.5% (v/v) acetic acid in water). Elution profile was 0 min 10% A in B, 28.6 min 60% A in B, 30 min 10% A in B. The flow rate was 1 mL/min, and the injection volume was 20 μl. By comparing the retention time of the eluted components to the retention time of the reference standard, the eluted components were recognized (system-controlled CMB-20A/20A liter). Phenolic compounds present in the samples were characterized according to their UV/VIS. Absorption was measured at 290 nm.

### DPPH radical scavenging activity

The *P. columbinus* extract 100 mg was mixed with 10 ml methanol (96%) and 63 μmoL/L of the alpha-diphenyl-beta-picrylhydrazyl (DPPH) solution to assess the fungal extract’s ability to scavenge the stable free radical DPPH. After different times (at 30, 60, and 90 min) at room temperature, the absorbance was measured at 517 nm and expressed as radical scavenging capacity according to Zeković et al. [10]. The radical scavenging capacity (RSC) was calculated using the following equation:

$$\mathrm{RSC}=\mathrm{Ac}-\mathrm{As}/\mathrm{Ac}\times 100$$

where Ac is the absorbance of the control; As is the absorbance of the sample.

### In vivo study

#### Experimental design

Wistar rats have been purchased from the laboratory animal colony. Thirty-two adults’ female Wistar rats, weighing 150‑180 g and aged 10‑13 weeks, were kept in the animal care facility. Rats were fed and watered according to the regular diet for rats with a 12-h light-dark cycle.

#### Chemicals

##### Cyclophosphamide (CTX)

The experimental CTX was represented as a vial of 200 mg from Baxter Oncology GmbH, Halle. The medication was dissolved in 10 ml of saline buffer phosphate.

#### Enhancement POF

Cyclophosphamide was implanted in rats by intraperitoneal injection (IP) at a rate of 50 mg/kg of body weight as a loading dose, followed by CTX IP (8 mg/kg) injection daily for 2 weeks [11].

#### Animals’ groups

We randomly assigned the animals into four groups: *group I:* control group (*n* = 8): received vehicle solution as 200 ppm saline; *group II*: CTX group (*n* = 8): injected with CTX as specified above; *group III:* normal rats treated with lyophilized TP (100 mg/kg) orally for two consecutive weeks. *Group IV* (curative group): CTX-TP (*n* = 8); this group received CTX (8 mg/kg body weight, IP) for 14 days and then they received TP (100 mg/kg) orally for two consecutive weeks, starting from the next day post CTX (from 15th to 28th of the experiment). We followed the guidelines of the Institutional Animal Ethics Committee regarding experimental research in small animals. Therefore, the rats were anesthetized by Na-pentobarbital (60 mg/kg i.p.) before blood was sampled.

#### Blood assays and tissue sampling

At the end of the experiment (29th day, the next day post TP), rats were subjected to anesthesia Na-pentobarbital (60 mg/kg i.p.), and an approximately 5 ml of blood samples were obtained from the orbital venous plexus in clean sealed tubes. Blood samples were centrifuged to sera at 3000×*g* for 10 min. The ovary was quickly excised, washed out with normal saline, blotted with filter paper, weighed, and put into ice-cold saline (0.9% NaCl). Approximately, 50‑100 mg of ovary tissue was hydrolyzed in 600 μL of lysis buffer saline (pH 7.4) with an Ultra-Turrax T-25 homogenizer (IKA®, Staufen, Germany). The homogenate was centrifuged at 10,000×*g* and 4 °C for 10 min, and the supernatant was used up for testing the oxidative stress enzymatic biomarkers.

#### Measurement of lipid peroxidation

The thiobarbituric acid reactant assay (TBARS) was estimated to detect lipid oxidation. Malondialdehyde (MDA), a split product of an endoperoxide from an unsaturated fatty acid resulting from the oxidation of lipid substrates. One hundred microliters of the samples were placed in microcentrifuge tubes. After that, 100 μl of SDS lysis solution was added to each microcentrifuge tube, which was then incubated at room temperature for 5 min. After the incubation, 250 μL of TBARS was added, followed by a 45–60 min re-incubation at 95 °C. All the samples were centrifuged at 554×*g* for 15 min after cooling in an ice bath for 5 min. The supernatants and MDA standards (200 μL) were placed in separate microplates, and the absorbance at 532 nm was measured.

#### Assessment of the oxidative stress enzymes

The ovarian antioxidant enzymes, including superoxide dismutase (SOD) [12], catalase (CAT) [13], and glutathione peroxidase (GPx) [14], were estimated using ELISA kits (Randox Laboratories, UK). The glutathione (GSH) was also assessed by the same protocol using commercial kits purchased from Northwest Life Science Specialties LLC (Washington, USA). Supernatant (from the ovarian samples that have been homogenized in PBS at 10000×*g* on the ice for 5 min) and the antioxidant standards (20 μL) were added into respective wells. Each samples and antioxidant standards were run in duplicate.

#### TNF-α serum measurement

A TNF-α sample level was measured using commercially available kits (Awareness Technology Inc., Chro Mate ELISA Reader; Cambridge, UK) by an enzyme-linked immunosorbent assay (ELISA). TNF-α levels were expressed in pg/ml.

#### Anti-Mullerian hormone (AMH) serum levels

The ELISA (Thermo Science Multiskan GO, Finland) was used to evaluate AMH (ng/mL) levels of the serum samples. The sensitivity of the analysis was recorded at 0.10 ng/mL assay with the test range of 0.3‑40 ng/mL.

#### Radioimmunoassay for serum LH, FSH, and E2

Homologous double-antibody radioimmunoassay techniques were used to quantify LH, FSH (100 μL of serum), and E2 in 50 μL of serum [15]. DI Asource ImmunoAssays S.A. (Rue de l’Industrie, B-1400 Nivelles, Belgium) was used to measure E2 (KIP0629), FSH (KIP0841), and LH (KIP1311) levels.

#### Analysis of Western blot

The immunoprobing of Western blots was used to measure the level of ovarian caspase-3 protein. The STE buffer (0.32 mol/L sucrose, 5 mmol/L tris, 2 μg/mL leupeptin), centrifuged at 4 °C, was made in 10 min, with 10% (w/v), and tissue homogenates (0.32 mol/L sucrose). The buffer was then centrifuged at 4 °C. In SDS sample buffers (50 mm Tris pH 7.5, 5% SDS, 5 mM DTT, 5% Glycerol), 6 M Urea was removed by centrifugation (14,000×*g*, 20 min), and the cell pellets were separated by heating (95 °C, 5 min), which included a sample buffer of SDS. Supernatants were tested with the Bio-Rad DC Protein Assay for protein content and processed until use at −80 °C. These protein extracts were transmitted to the nitrocellulose membranes (Amersham Life Science, Little Chalfont, UK) utilizing a system of mini-protein electrophoresis (30 V, 16 h). Membranes were blocked overnight at raw fiber with 3% (w/v) bovine serum albumin (BSA) in 0.1% (v/v) Tween 20 TBS (TTBS) before being probed for 2 h at room temperature with a polyclonal rabbit anti-rat caspase-3 (Santa Cruz Biochemicals, USA) at 1:2000 dilutions in TTBS buffer. An anti-rabbit peroxidase conjugate was diluted at 1:2000 in TTBS buffer for 1 h. Then, the ECL chemiluminescent detection system was used to reveal primary antibody binding (Life Science). Recombinant Caspase-3 was used to test the antibody effectiveness under experimental circumstances (Sigma) canning densitometers of the Bio-Rad GS-690. The software versions 4 Molecular Analyst were assessed for the volume density (Bio-Rad Laboratories Ltd., Hertfordshire, UK). Compared with protein molecular weight markers (Bio-Rad Laboratories Ltd.), the translation size was calculated using the same analysis tool.

#### DNA fragmentation by agarose gel electrophoresis

Ovarian tissues (10 mg) were homogenized from the animal in each group and used for DNA isolation by PBS-E homogenization (50 mM sodium phosphate buffer containing 0.9% saline and 20 mM EDTA, pH 8). DNA was suspended in 2 mL PBS-E with a collagenase of 0.5 mg/ml. The Ultra Turrax homogenizer (IKA T 25-German) was employed, and ice testing specimens before and after homogenization were maintained. First, a stirring accompanied by a pronase E addition (1 mg/mL) for 1 h was incubated with a stirring at 37 °C and then incubated for 15 min at 37 °C. Then, the suspension was centrifuged at 1000×*g* for 5 min. Next, a pellet with 2 ml lysis buffer containing 50 m MTris-HCl, pH 8, 20 mM EDTA, 10 M NaCl, and 1% (w/v) SDS were dispersed and infused and again centrifuged for 15 min, at 14,000×*g*, with phenol-chloroform extraction [16] of the pellet extracted from the lysate. Next, at 65 °C at 10 mM Tris-HCl, pH 8, containing 1mM EDTA, DNA was dissolved by gentle shaking. Next, 2% agarose gel was prepared as 1 g of agarose was dissolved in 50 mL of Tris-Acetate EDTA (TAE) buffer in a flask covered with an aluminum sheet for 5 min microwave adjustable to medium temperature for the study of DNA fragmentation. In addition, 2.5 μl of ethidium bromide (EB) was added to the agarose solution chilled to 60 °C to allow the visualization of DNA. Gloves were required in dealing with EB dye solutions because it is a highly sensitive mutagen. The gel was spilt into a pure, dry gel mold. The comb was gently eliminated, and the gel was placed in the electrophoresis chamber when the agarose had set (20–30 min).

#### Histological examination

Hematoxylin and eosin (H&E) staining was performed to test for ovarian damage by Bova et al. [17]. Ovarian samples from all groups were fixed in 10% formalin for 48 h, and paraffin blocks were prepared. Each sample was cut at 5 μM, and the slides were covered with poly-lysine. Then, paraffin was removed from each slide in xylene, hydrated with different downward concentrations of ethanol, and stained in hematoxylin. Then, the slides were stained from the eosin counter, dried, and placed in increasing concentrations of ethanol and xylene. Pictures were taken under a microscope. Ovarian follicles were counted in each slide, according to Maciel et al. [18] with the help of the Leica Qwin 500 LTD image analysis software (Cambridge, UK).

#### Smear of vagina

In the early morning, vaginal secretion was obtained by injecting a plastic pipette filled with 10 μl of regular saline (0.9% NaCl) into the vagina of the rat, using the presence of cornified cells as an indication of estrogenic activity [19].

### Statistical analysis

The findings were expressed as mean ± SEM. For statistical analysis, one-way analysis of variance accompanied by using the Tukey Multiple Comparison tests as a post hoc test was used, where *P* < 0.05 was the agreed standard of statistical significance.

## Results

The results illustrated that the mushroom *P. columbinus* was rich in carbohydrates and protein (43.0% and 17.0%, respectively) but low in fiber and fat (2.5% and 4.5% DW, respectively) with an ash content of 5.7% DW.

### Total phenolics and flavonoids compounds

The amount of 28.13 mg/g gallic acid equivalent/mL of extract was found. The total phenolic and total flavonoid literature of mushroom were low compared to previous data from literature of *Pleurotus* species (45.6 mg/g). The total flavonoids content of *P. columbinus* was observed as 6.6 mg quercetin equivalent/g of extract.

### Phenolic compound identification by HPLC

**Fig. 1:**
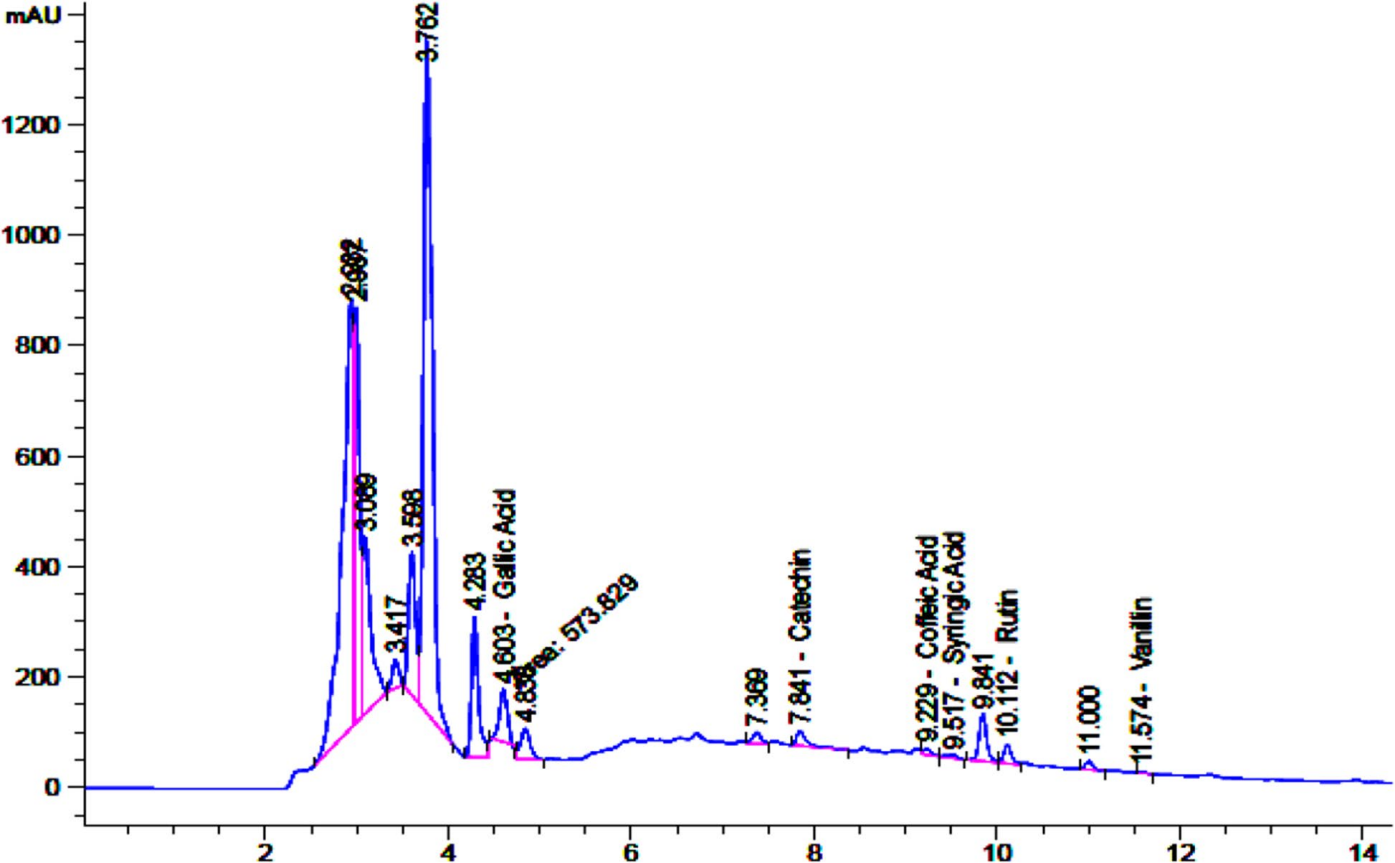
HPLC chromatogram recorded at 290 nm of extract, peaks related to phenolic compounds

Phenolic compounds include different subclasses, and they displayed a large diversity of structure. We found catechin (32.77 μg/mL), gallic acid (21.86 μg/mL), rutin (13.34 μg/mL), vanillin (0.23 μg/mL), syringic acid (1.64 μg/mL), and coffeic acid (1.61 μg/mL) are present in the sample at retention times 7.84, 4.60, 10.11, 11.57, 9.51, and 9.22 min respectively. These compounds were identified (Fig. 1) from TP by comparing their chromatographic characteristics with the standards compounds.

### DPPH radical scavenging activity

The present study proved the DPPH scavenging activity of phenolic extract at different times 0, 30, 60, and 90 min. The antioxidant activity was increased with increasing time. The maximum scavenging activity (90.21%) at 0.1 mg/mL was observed after 90 min.

### In vivo results

#### Body, ovarian, and uterine weight changes

After 14 days of administered CTX, the rats lost approximately 1.28-fold their body weight compared to the control group 182 ± 8.13 and 142.22 ± 5.40 g, respectively (Table 1). Likewise, the same effect happened on the ovarian and uterine weight, where they lost about 2.02 and 1.92-folds, respectively, compared to the control group (Table 1). Moreover, a significant increase in CTX-treated groups and significant depression in both groups receiving TP before and after CTX in body weight amount 1.33-fold also ovary and uterine wet weight amount to 1.73 and 1.56-folds, respectively compared to the CTX group (Table 1). Thus, although ovarian and uterine weights were increased in the mushroom extract-treated group and were significantly higher than in the CTX group, they were still somewhat lower than in the control group.

**Table 1:** The antagonistic effect of *P. columbinus* extract in alleviating the toxic effect of CTX on body weight, ovary, and uterine weights of rats

Groups	Body weight (g)	Ovarian weight (mg)	Uterine weight (mg)
Control	182 ± 8.13^b^	88.16 ± 6.75^a^	119.33 ± 10.11^a^
CTX	142.22 ± 5.40^c^	43.70 ± 3.54^c^	62.15 ± 8.32^c^
*P. columbinus* extract	204.32 ± 11.52^a^	84.67 ± 5.68^a^	112.72 ± 9.67^a^
CTX + *P. columbinus*	189.17 ± 9.04^bc^	75.81 ± 5.43^b^	97.17 ± 7.36^b^

Each value represents mean ± SE (*n* = 8). a, b, ab, and c denote significant difference from normal-control, CTX-induced POF, *P. columbinus*extract control and POF*+P. columbinus* (before and after) groups, respectively, *p* < 0.05 (one-way ANOVA followed by post hoc Tukey)

#### Serum hormones evaluation

Table 2 shows that the CTX group had early ovarian failure resulting in serum AMH and E2 levels of 2.45 and 2.77-folds lower than the control values, respectively. Likewise, LH levels were approximately 2.32-folds lower than the control group in rats with premature ovarian failure. However, the results recorded a remarkable increase in these parameter treatments with mushroom extract (Table 2). Regarding the level of FSH, there was a significant (*p* < 0.05) increase by 1.59-fold (6.65 ± 0.18) in the CTX animals after 14 when compared to the control group (4.17 ± 0.07) (Table 2). However, administrations of the mushroom extract improved the level of FSH with values of 4.75 ± 0.31 (28.60%) lower than the CTX group and are close to the control values.

**Table 2:** The antagonistic effect of *P. columbinus* extract in alleviating the toxic effect of CTX on serum hormone levels in the experimental rats

Groups	E2 (pg/ml)	AMH (ng/ml/)	FSH (mIU/ml)	LH (mIU/ml)
Control	11.05 ± 0.78^a^	7.10 ± 0.50^a^	4.17 ± 0.07^bc^	1.30 ± 0.02^a^
CTX	4.51 ± 0.33^b^	2.56 ± 0.04^b^	6.65 ± 0.18^a^	0.56 ± 0.01^c^
TP	12.13 ± 1.35^a^	7.16 ± 0.62^a^	3.93 ± 0.06^c^	1.27 ± 0.007^ab^
CTX+ TP	12.30 ± 1.58^a^	6.30 ± 0.41^a^	4.75 ± 0.31^b^	1.14 ± 0.02^b^

Each value represents mean ± SE (*n* = 8). a, b, ab, and c denote significant difference from normal-control, CTX induced POF, *P. columbines* extract control and POF*+P. columbinus* (before and after) groups, respectively, *p* < 0.05 (one-way ANOVA followed by post hoc Tukey)

#### The effect of the mushroom extract on ovarian antioxidants, TBRAs, and TNF-α

The improving role of mushroom extract in POF induced by exposure to CTX was evident in the ovarian antioxidant enzymatic activities and the lipid peroxidation represented by TBARS (Table 3). A significant (*p* < 0.05) decrease in the levels of SOD, CAT, GSH, and GSH-PX were observed after receiving CTX. These reductions in the antioxidants were 2.25, 2.43, 2.29 and 2.39-fold, respectively, compared to the controls. In addition, the decrease in the antioxidants was associated with a significant increase in TBRAs (*p* <0.05, 4.57-fold) compared to the control rats. However, administration of the mushroom extract significantly (*p* < 0.05) improved concentrations of these defensive enzymes and decreased TBRA levels (Table 3).

**Table 3:** The antagonistic effect of *P. columbinus* extract in alleviating the toxic effect of CTX on oxidative stress markers in the experimental rat groups

Groups	TBRAS (nmol/ml)	CAT (U/g)	SOD (U/g)	GSH (U/mg)	GSH PX (U/g)
Control	0.37 ± 0.02^b^	14.70 ± 1.67^a^	84.03 ± 6.09^a^	10.27 ± 0.81^a^	10.23 ± 0.56^a^
CTX	1.69 ± 0.05^a^	6.05 ± 0.54^b^	37.33 ± 3.41^b^	4.48 ± 0.33^b^	4.28 ± 0.06^b^
TP	0.39 ± 0.03^b^	14.13 ± 1.32^a^	90.70 ± 5.23^a^	10.02 ± 0.63^a^	10.01 ± 0.71^a^
CTX+TP	0.54 ± 0.04^b^	13.90 ± 1.12^a^	75.83 ± 3.44^a^	8.92 ± 0.41^a^	9.993 ± 0.56^a^

Each value represents mean ± SE (*n* = 8). a and b denote significant difference from normal control, CTX induced POF, *P. columbines* extract control and POF*+P. columbinus* (before and after) groups, respectively, *p* < 0.05 (one-way ANOVA followed by post hoc Tukey)

**Fig. 2:**
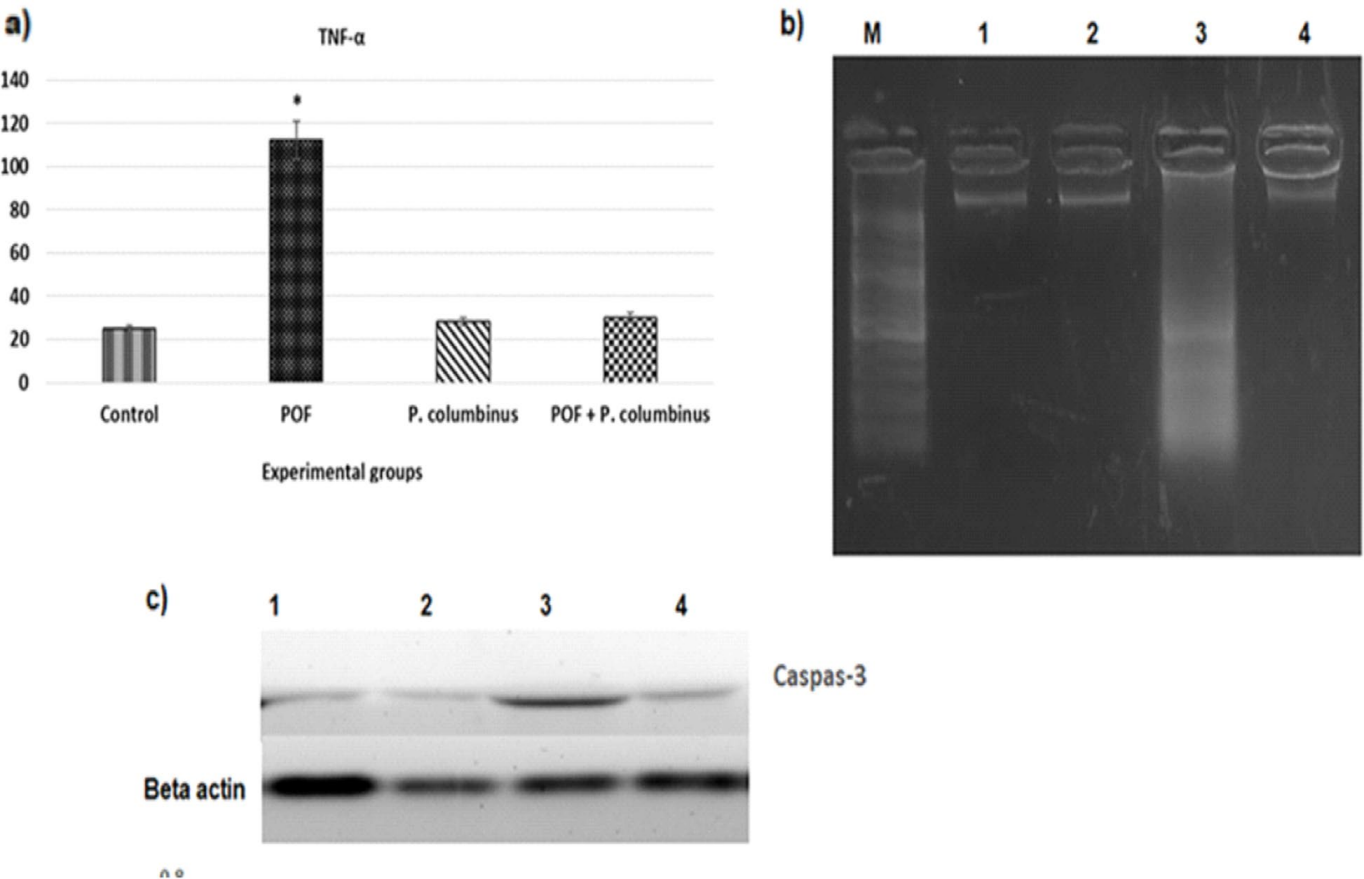
**a** Oral treatment of *P. columbinus* extract (100 mg/kg) on TNF-α in rat treated with CTX chemotherapy. Each bar is represented by a mean ± SE (*n* = 8). Asterisk indicates a significant difference from the control and treated with *P. columbinus* extract. **b** Agarose gel electrophoresis shows DNA fragmentation. Lane 1: M: DNA marker with 100 bp. Lane 2: control group. Lane 3: normal rats administered *P. columbinus* extract. Lane 3: POF model. Lane 4: CTX+*P. columbinus* extract. **c** Western blot analysis for protein expression of caspase-3 and beta actin in different studied groups. Lane 1: control group. Lane 2: normal rats administered *P. columbinus* extract. Lane 3: POF group. Lane 4: CTX+ *P. columbinus* extract. Data is expressed as mean ± SD of three independent experiments. *p < 0.05 ≠ control and *P. columbinus* extract groups

Figure 2a shows the effect of CTX and treatment with TP in rats. Using chemotherapy resulted in a significant increment in the TNF-α by about 4.47-fold compared to the control group. The administration with TP at a 100 mg/kg concentration after 14 days showed the ameliorative effect in TNF-a. It led to a notable decrease of ~ 3.72-fold compared to the CTX group that did not receive the mushroom extract (TP).

#### Ovarian DNA and caspase-3

The harmful apoptotic effects of the animal rats exposed to CTX were mainly detected in this study using the DNA fragmentation technique (Fig. 2b). The DNA ladder depicted a sequence of fragments that are multiples of 180~200 bp. Compared to the control, the diffuse pattern of the DNA fragments in the CTX was distinct. The current study showed a marked rise in the CTX group of 5.14 folds compared with controls in the case assessment of caspase-3 (Fig. 2c). Contrarily, there was a marked amendment in DNA fragmentation after treatments with the different concentrations of *P. columbinus* extract (Fig. 2b).

#### Histopathological findings

**Fig. 3:**
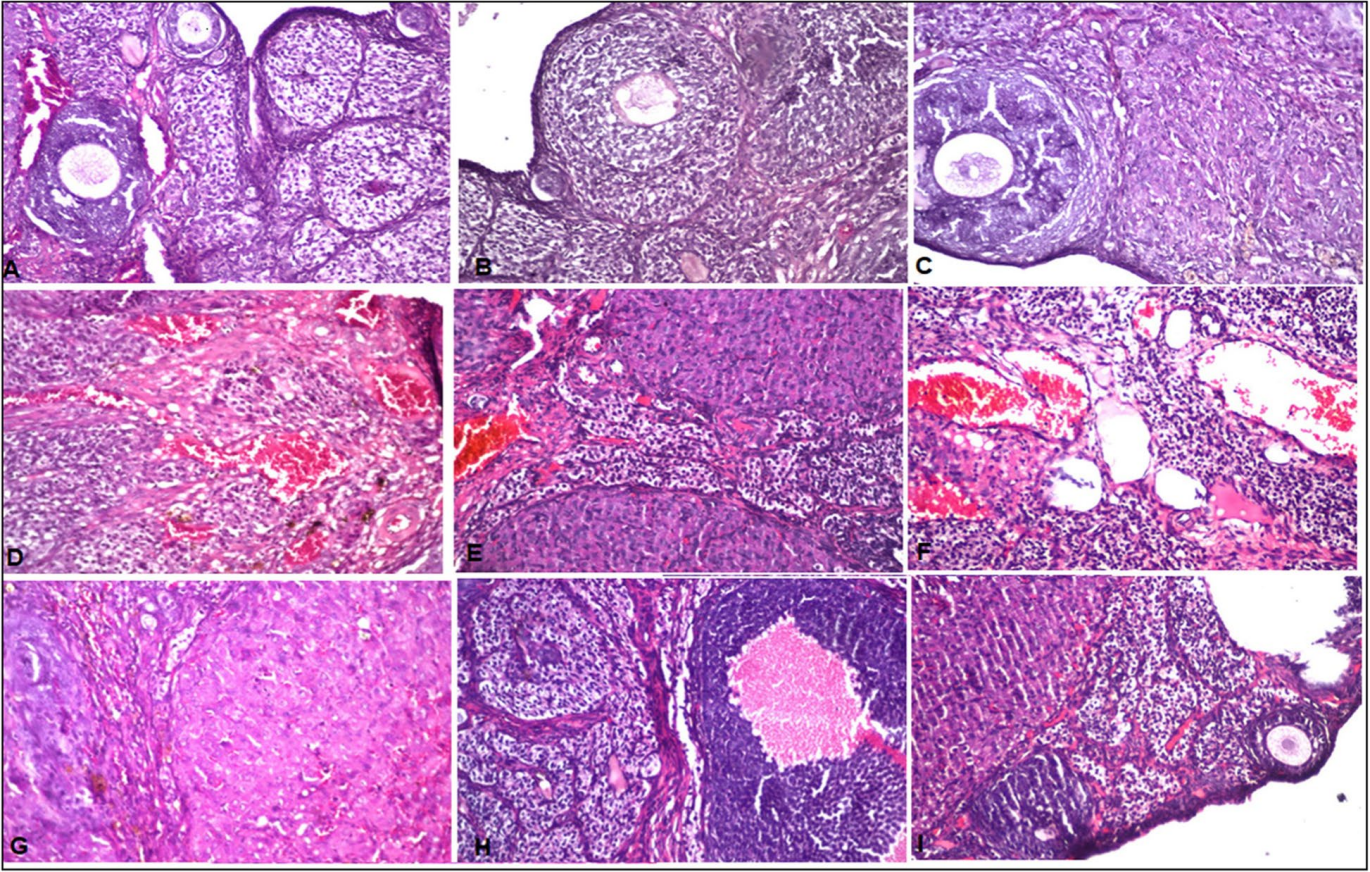
**a** Control group: Interstitial stromal cells with primary and secondary follicles and few corpus luteum were detected (**a** and **b**). **c** Control rats received *P. columbinus* extract; there is no alteration in the primary and secondary follicles. **d** Group of rats injected CTX: Sever congestion was detected in the medullary blood vessels associated with mature follicle and corpus luteum in the cortex. There was congestion in the medullary portion associated with few atretic follicles as well as multiple corpus luteum (**e**). There was multiple corpus luteum with few follicles as well as interstitial stromal cells in the cortex. Sever congestion was detected in the medullary blood vessels. There was multiple corpus luteum with few follicles as well as interstitial stromal cells in the cortex (**e** and **f**). Sever congestion was detected in the medullary blood vessels (**g**). (**h** and **i**) CTX+ *P. columbinus* extract (100 mg/kg): Interstitial stromal cells with normal primary and secondary follicles. **j** Oral treatment of *P. columbinus* extract (100 mg/kg) on the number of follicles in rats treated with CTX chemotherapy

The treatment of TP has reinforced the improvement of ovarian follicular failure caused by CTX, as shown in Fig. 3 h and i.Several rising follicles (primordial, preantral, and antral) and ordinary granulosa cell layers (Fig. 3h and i) at various stages. It is noteworthy that the number of follicles and the primary, mature, and secondary follicles in the CTX group became fewer than the control group by about 2.44, 2.47, and 4.28-folds, respectively (Fig. 3j). Treatment with TP worked to overcome the effect of chemotherapy on the number of follicles in their different stages. The numbers of primary mature and secondary follicles increased by about 2.34, 2.03, and 3.37-folds, respectively, compared to those left untreated.

#### Vaginal smear

In the postoperational cycle (100% frequency), all animals displayed estrogen activity at the end of TP treatment (GIII and GIV).

## Discussion

The main contents of TP are the total carbohydrate, total protein, total lipid, crude fiber, and ash content. Daba et al. [20] reported that carbohydrates represent the essential constituent in *Pleurotus* species ranging from 50‑80%. The protein content in the analyzed mushroom was 16.99%. This value is between mushrooms from Chihuahua, Mexico (12.95‑33.73%) [20]. In *Pleurotus florida*, the total carbohydrates, fat, protein, fiber, and ash were 32.08, 1.54, 27.12, 23.18 and 9.41%, respectively.

The mushroom has a significant nutritional value, with high protein concentration and a small quantity of total fat produced by its constituents [20]. Strain, growth media composition, harvest timing, management strategies, handling circumstances, and substrate preparation were all factors affecting mushroom characterizations [21]. The findings were like analysis by Bellettini et al. [22].

### Total phenols

Phenolic compounds are a substantial number of plant secondary metabolites with antioxidant properties that can act as free radical scavengers, hydrogen donors, and singlet oxygen quenchers, all of which are important in the redox reaction’s defensive strategy. Many studies have shown that mushrooms have scavenging potential, farther they contain many phenolics that are indispensable plant constituents [23]. Phenolics also have a considerable variety of biological effects, including antibacterial, anti-inflammatory, and antihyperglycemic effects [24]. In this study, the amount of phenolic content of mushroom was the difference could be attributed to genetic factors, extraction time, temperature, and type of solvent.

### Total flavonoids

Rengasamy et al. [25] reported that flavonoids are phenolic compounds that make up the most prevalent distributed phenolic group in plants. Besides, anthocyanidins, chalcones, flavanols, flavanones, flavones, flavanols, and isoflavone are major flavonoid classes. The total flavonoid content (TFC) varies depending on the mushroom species. The results were comparable to those reported by Palacios et al. [26], who declared that, among eight edible mushrooms, *Lactarius deliciosus* had a higher amount of total flavonoids. Besides, diversified values of TFC were found to have higher levels of flavonoids than the other species in five edible mushroom species, including *Agaricus sivaticus* [27].

### Antioxidant activities of TP

The phenols are one of the considerable contributors to the antioxidant activity of different mushrooms [28]. Its ability to impart a hydrogen atom or an electron represented by its captive action of the DPPH-free radical measured the antioxidant activities of the plant extract used in our study, as diphenyl-2-picryl-hydrazine, stable free radicals. In comparison with the antioxidant ability of various extracts of edible mushrooms (Pleurotus), these findings can be regarded as high antioxidant capacity [29]. Aryal et al. [30] suggested that phenolic and flavonoid groups are highly responsible for the antioxidant activity of the selected plant extract, not one component of them due to correlation of total phenolic and flavonoid content with antioxidant capacity. We need further studies to recognize that the phenolic compounds take the antioxidant activities and the mechanism of the phenolic compounds as an antioxidant. Also, in vivo antioxidant assays are required to confirm the potential use of this phenolic extract of mushrooms in the therapy of different diseases.

### In vivo findings

The serum E2 level decreased in this post-CTX sample, correlated with a substantial increase in FSH compared to the healthy group, and these data show the existence of standard POF. Our findings were compatible with those of the previous study [31], which had proved this. The administration of TP substantially increased the hormonal changes resulting from cyclophosphamide intake used to treat cancer.

Our findings were compatible with those of the previous study [32]. By destroying ovarian tissue, chemotherapy has a detrimental effect on the ovaries.

A study had previously established that cyclophosphamide destroyed primordial follicles in proportion to the increased dose [33]. Besides, AMH was assessed in the various treatment groups because the evaluations of FSH and E2 could not express the capacity of the ovary to provide egg cells and fertilization before infertility development. The anti-Mullerian hormone is a hormonal protein that is produced by follicle cells [34]. Serum AMH has been suggested as a biomarker for ovarian syndrome diagnosis since it corresponds with the total number of antral follicles on both ovaries. These findings were consistent with human and experimental studies that previously recorded depressed AMH levels after chemotherapy or radiation [33].

Interestingly, TP approximately overwhelmed these faults caused by 14-day chemotherapy. The higher FSH levels reported after gonadotoxic treatment revealed a decline in ovarian function, consistent with reduced AMH levels [35]. In rats undergoing CTX therapy, we also evaluated different follicular numbers. In contrast to the control group, substantial depletion of the primordial follicle pool was observed. Our findings were consistent with a previous study that showed that primordial follicles are recruited at a more accelerated rate in the absence of AMH, resulting in premature depletion of the primordial follicle population [30].

Interestingly, in rats treated with *P. columbinus* extract, ovarian vesicle clusters improved compared to rats treated with chemotherapy (POF). Two pathways followed adverse effects caused by CTX; direct and indirect [32]. The direct approach is happened through DNA ionization, while the indirect process generated reactive oxygen species (ROS). In the present study, the assessment of ovarian DNA fragmentation and caspase-3 revealed a significant increase cleaved in CTX rats compared to the control group.

Results from a previous study showed that CTX toxicity increased ovarian tissue lipid oxidation and TBRA levels. These results were interpreted based on the principle of free radical formation during chemotherapy treatment due to leakage of electrons from the inner mitochondrial membrane during oxidative phosphorylation and generation of steroid ATP [36]. Oxidative stress disrupted cellular redox circuits, leading to disturbances in the redox-regulating cellular processes. Therefore, these free radicals interact with cellular lipids, proteins, and nucleic acids, thus preventing the development of H_2_O_2_ steroids in ovarian cells, eventually leading to ovary failure at various stages to shape its cells and follicles [37].

In our study, the GSH, GSH-PX, CAT, and SOD activities in the ovaries were significantly depleted. Besides, lipid peroxides were significantly increased after receiving CTX. However, the administration of TP for 14 days could improve antioxidant levels compared to groups with early ovarian failure from chemotherapy. Furthermore, a recent study has shown that resistance to the toxic effect of chemotherapy by modifying antioxidants [33]. Therefore, our research illustrated that countering oxidative stress and a lipid peroxidation is an extra tool through which TP can preserve the CTX-induced ovarian follicle failure.

## Conclusions

The data of this study showed the chemical structure of *P. columbinus* extract and its containment of total phenols and flavonoids. Thus, these compounds improved the experimentally induced premature ovarian failure syndrome in female rats through their role as antioxidants and their effect on female sex hormones. The histological study of the ovaries also confirmed these biochemical results, as the administration of mushroom extract led to a clear improvement of ovarian tissue. Therefore, through experience, the efficacy of the TP is explicit in the recovery from early ovarian failure. We recommend conducting future clinical trials on this fungus because of its nutritional and therapeutic importance. TP may be expected as a possible promising medicinal for ameliorating the POF and its related oxidative damages. Subsequently, encouraging health-beneficial effects makes it a potentially valuable natural candidate for the pharmaceutical industries worthy of further research in this area. The examination of bioactive components in edible mushrooms is still insufficient. Mushrooms with nutraceutical and health benefits are given various potential characteristics, which deserve further investigation.

## Data Availability

The data is available and it will be provided to the editor if required.

## Abbreviations

CTX: Cyclophosphamide
AMH: Anti-Mullerian hormone
LH: Luteinizing hormone
FSH: Follicle-stimulating hormone
TBARS: Thiobarbituric acid reactant assay
MPA: Malondialdehyde
TBA: Thiobarbituric acid
GPx: Glutathione peroxidase
SOD: Superoxide dismutase
CAT: Catalase
LLC: Life Science Specialties
DW: Dry weight
IP: Intraperitoneal injection
DPPH: 1,1-Diphenyl-2-picrylhydrazyl
PBS: Phosphate buffer solution
TNF: Tumor necrosis factor
TP: Total phenol
